# Reducing education inequalities through cloud-enabled live-cell biotechnology

**DOI:** 10.1016/j.tibtech.2024.07.015

**Published:** 2024-08-28

**Authors:** Samira Vera-Choqqueccota, Baha Eddine Youcef Belmekki, Mohamed-Slim Alouini, Mircea Teodorescu, David Haussler, Mohammed A. Mostajo-Radji

**Affiliations:** 1 Live Cell Biotechnology Discovery Laboratory, University of California Santa Cruz, Santa Cruz, CA 95060, USA; 2 Genomics Institute, University of California Santa Cruz, Santa Cruz, CA 95060, USA; 3 Department of Biomolecular Engineering, University of California Santa Cruz, Santa Cruz, CA 95060, USA; 4 Computer, Electrical, and Mathematical Sciences and Engineering (CEMSE) Division, King Abdullah University of Science and Technology (KAUST), Thuwal 23955-6900, Kingdom of Saudi Arabia; 5 Department of Electrical and Computer Engineering, University of California Santa Cruz, Santa Cruz, CA 95060, USA

## Abstract

Biotechnology holds the potential to drive innovations across various fields from agriculture to medicine. However, despite numerous interventions, biotechnology education remains highly unequal worldwide. Historically, the high costs and potential exposure to hazardous materials have hindered biotechnology education. Integration of cloud technologies into classrooms has emerged as an alternative solution that is already enabling biotechnology experiments to reach thousands of students globally. We describe several innovations that collectively facilitate real-time experimentation in biotechnology education in remote locations. These advances enable remote access to scientific data and live experiments, promote collaborative research, and ensure educational inclusivity. We propose cloud-enabled live-cell biotechnology as a mechanism for reducing inequalities in biotechnology education and promoting sustainable development.

## Towards inclusive and equitable biotechnology education

The United Nations Sustainable Development Goals (SDGs) prioritize inclusive and equitable education globally [1]. However, access to quality education remains unequal, particularly in the sciences [2,3]. Inadequate monitoring and assessment systems frequently hinder policy development [1,4]. Achieving equitable biotechnology education for all will impact on other SDGs, including poverty eradication, food security, health, gender equality, clean water and sanitation, and sustainable energy [5–7].

Biotechnology education is most effective when it includes hands-on projects focusing on trending and relevant topics [8,9]. However, implementing education modules is limited by at least three roadblocks: (i) advanced training for instructors, (ii) specialized biotechnology equipment, and (iii) potential exposure to hazardous materials [10]. The integration of cloud technology into biotechnology education emerges as a key innovation to overcome these roadblocks owing to the scalability, flexibility, and efficiency of the technology [11]. Cloud technology facilitates practical, hands-on experiences into a theoretical curriculum by enabling students to perform experiments remotely. Through this approach, students can acquire novel skills, make new discoveries, and collaborate [12].

‘Cloud laboratories’, in which benchtop equipment is operated over the internet, have enabled the collaborative study of cells, tissues, and organisms. This approach is driving several multinational initiatives, including drug synthesis [13], protein engineering [14], and brain observatories [15]. We define cloud-enabled live-cell biotechnology as the ability to observe, analyze, and manipulate living tissue remotely. In biotechnology education, the use of the cloud makes inquiry-based education possible, reaching a scale comparable to that of massive open online courses (MOOCs) [16–20].

**Figure 1.**
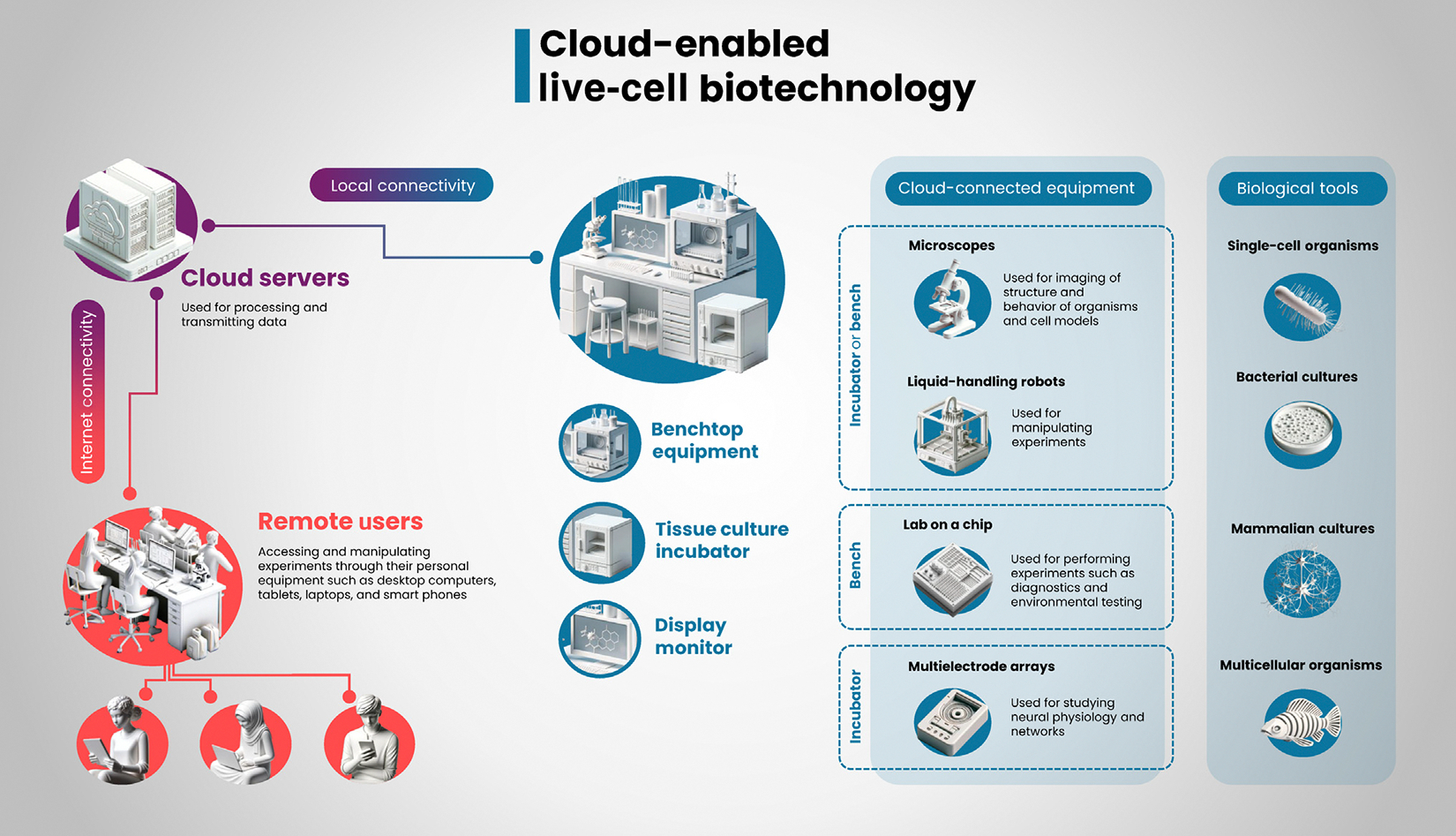
Overview of cloud-enabled live-cell biotechnology. Cloud connectivity enables students worldwide to remotely access laboratory equipment, including benchtop instruments and devices inside tissue culture incubators, such as microscopes, liquid-handling robots, laboratory-on-a-chip technologies, and multielectrode arrays. A diverse array of biological tools are utilized, ranging from single-cell organisms and microorganism cultures to mammalian cell cultures and small multicellular organisms.

To increase the reach of live-cell biotechnology globally, several innovations need to converge to enable the use of these technologies in the classroom, particularly in underserved regions of the world (Figure 1). We review these technologies, including the development of low-cost cloud laboratory equipment and educational modules that leverage complex biological models. We propose that the complementation of these technologies with new pedagogical tools and scalable options for internet access can transform the educational landscape and accelerate the SDGs.

## Leaving no one behind (LONB)

The LONB principle is central to the SDG agenda, and access to internet-based education has been shown to improve academic performance [21]. However, 46.4% of the world population is currently unconnected to the internet [22], including 1.3 billion children aged 3–17 years [23].

Traditionally, two structural roadblocks have hampered internet connectivity: lack of infrastructure and limited affordability of the technologies [22]. We propose two additional social-based roadblocks of equal importance for LONB: digital illiteracy and legal limitations.

### Lack of infrastructure

The majority of the offline population lives in areas of the world at least partially covered by 3G and 4G network connectivity [22]. However, ~11% of the population lives in regions completely unconnected [22]. These populations are often in low-density areas in the developing world, where traditional communications systems are economically unfeasible [22]. In the developed world, these populations are in rural and quasi-rural regions, which leaves these people isolated from regional conversations, decision making, and education [24].

### Limited affordability

Even when the internet is available, high costs may make it inaccessible. In the developed world, internet affordability represents an issue of racial equity because Black and Latinx neighborhoods often have lower adoption rates [25]. In these situations, community infrastructures, such as libraries, can help to fill the gap. In the developing world, where community infrastructures are lacking, the adoption of low-cost mobile internet devices [26] can become the bridge towards cloud-based education.

### Digital illiteracy

Digital illiteracy is disproportionately observed in the elderly and the poor [27]. Simplifying interfaces that enable human–computer interactions (HCIs) can make the adoption of cloud biotechnology feasible for these vulnerable populations. Addressing digital literacy through targeted education programs can further support these groups.

### Legal limitations

The majority of the world population lives in countries with intermediate or high internet censorship [28]. In addition, there are non-negligible populations in restricted areas, such as the 10 million people currently incarcerated worldwide who could benefit from cloud-based education [29]. People living near to radio quiet zones also face unique challenges regarding internet access.

By addressing these roadblocks, cloud-based biotechnology education can adhere to the LONB principle and contribute to the equitable dissemination of knowledge.

## Cloud-enabled laboratory hardware

Cloud-connected laboratory devices encompass a broad range of instruments that are essential for the practice of biology. They include microscopes, liquid-handling robots, laboratory-on-a-chip (LoC) systems, and electrophysiology setups.

### Microscopy

Although several commercially available cloud-enabled microscopes are available on the market, they are prohibitively expensive for educational settings. Therefore, biotechnology education programs have benefited from the use of 3D printing technologies and low-cost off-the-shelf components.

Early work in cloud-enabled microscopy focused on simple benchtop experiments using single-camera microscopes [18]. In this approach, the microscopes were developed using fixed objectives and streaming cameras. Users controlled the camera on/off switch, the microscope light, and the focal plane [18], and the systems streamed from a local router to a custom-made server [30].

Implementing these systems enabled remote experimentation in high school, undergraduate, and graduate courses using biological specimens [17,18]. An early iteration of this work used the slime mold in an education module of a graduate-level biophysics course to teach multicellular biological pattern formation to students without a previous biology background [17]. Although this course was small (four students), it provided the first proof of principle of the feasibility of using cloud-controlled experiments in the classroom. During the course the students discovered a previously unreported random self-avoidance pattern in the slime mold [17].

A second level of innovation stemmed from the integration of cloud microscopes and polydimethylsiloxane (PDMS)-based microfluidic chips [18]. These chips contained *Euglena gracilis* cultures and featured four light-emitting diodes (LEDs) [18]. These modifications allowed users to control the illumination intensity and duration to examine the phototactic behaviors of *E. gracilis* [18]. The data were then annotated by a custom-made interface [18]. Making a single module, the experiment was repeated >2300 times [19,31]. These experiments showed that live cloud-enabled biology experiments could be performed at a large scale in an educational setting. However, this scalability came with the cost of standardizing the experiment such that it could be repeated by more students without increasing the operational costs [19,31].

A different approach has been to design context-aware microscopy modules that focus on issues relevant to the students [16,32]. This approach has been shown to be effective at transmitting knowledge and developing science, technology, engineering, and mathematics (STEM) identity in students from under-represented backgrounds [8,33–35]. Two different cloud-enabled microscopes have been used in education: the Picroscope [36,37] and the Streamscope [16,32].

The Picroscope is a low-cost brightfield microscope consisting of an array of 24 cameras mounted onto focal length lenses with *z*-stack capabilities [36]. Each camera is controlled by a Raspberry Pi computer and transmits information via WiFi [36]. It functions both on the benchtop and inside tissue culture incubators. The microscope is primarily assembled from 3D printed materials and off-the-shelf components. All 3D printing is done using black polylactic acid (PLA) to reduce background illumination.

Several education programs have been performed using the Picroscope, ranging from the integration of remote microscopy projects into the biology high school curriculum to after-class college-level programs [16]. To date, the Picroscope has reached hundreds of underserved students in seven countries on three continents [16]. Projects have included survival and biocompatibility assays, drug screening, and developmental biology studies [6,35]. For example, students in Latin America performed a toxicity study of chlorine dioxide [16], a chemical that was promoted by pseudoscientific groups as a treatment for COVID-19 [38,39]. In addition high school students in the agricultural community of Salinas, California, performed drug screens on cells derived from neuroblastomas [16], a common health problem in agricultural regions [40]. Using the Picroscope, Salinas students also discovered that exposure to even low levels of ammonium nitrate delayed, but did not eliminate, fin formation in late-stage zebrafish embryos [16], which complemented the scientific literature.

Similar to the Picroscope, the Streamscope is an array of cameras mounted on lenses designed to work both outside and inside tissue culture incubators [16,32]. It captures images in brightfield and has *z*-stack capabilities. It can directly output information to common streaming platforms, including YouTube [32], thereby simplifying the user experience. The Streamscope has been used to introduce students to drug screening experiments using brain organoids [32]. Given the steadily increasing demand for organoid culturing skills in the biotechnology sector [41], the incorporation of cloud-enabled organoid experiments can become a powerful tool to train the next generation of biotechnology professionals.

### Liquid-handling robots

The ability to manipulate experiments remotely through liquid-handling robots has often complemented microscopy-based courses [17,20,42]. The majority of approaches have used low-cost materials combined with Raspberry Pi computers with WiFi capabilities [17,43]. Early work used LEGO bricks to design a gantry for positioning a syringe in coordinates determined by the user [17,44]. A LEGO-based actuator was then used to deliver liquid volumes [17,44].

Newer alternatives have been developed to enable users to have more flexibility in the experiments. One is the EvoBot, which has been built from off-the-shelf components and laser-cut parts [45]. It consists of an experimental layer that can hold experiments and an actuation layer which is modular and handles syringe, pump-based, and heavier payload modules and can inject liquids with 100 μl precision [43]. The EvoBot has a cloud-based interface that enables users to control the operations of the liquid-handling robot remotely [46].

OpenLH, on the other hand, takes advantage of commercially available robotic arms and complements them with custom-made liquid-handling attachments [47]. The attachments have included syringe pumps and devices made of spare parts of micropipettes [47]. A custom interface enables users to manipulate the OpenLH remotely [47]. Several experiments have been carried out, including teaching serial dilutions and targeting visual designers using bacteria as a biological ink to produce art [47].

### Laboratory-on-a-chip

LoC technology miniaturizes and consolidates various laboratory functions onto a single chip, typically only a few square centimeters in size. LoCs have been used in several biotechnology areas such as diagnostics [48,49] and environmental studies [50]. LoCs use microfluidics for pumping, mixing, separating, and dispensing liquids [51].

LoCs have been used in chemistry, physics, and bioengineering courses to engage students in engineering design and to introduce them to complex microfluidics concepts [52–55]. However, all these courses were conducted in person. Integrating LoCs with cloud technologies enables remote learning opportunities that now also include bioinformatics and other biological fields [56]. For example, biotechnology undergraduate students in Bolivia used a remote LoC device that integrates microfluidics, optical detection, and internet-based control to learn programming while assessing water quality. The devices incorporated pneumatically controlled valves for precise fluid manipulation and used DNA dyes to detect bacteria in the samples. The students, who had no computer programming experience, were challenged to complete and execute code for staining and detecting bacteria contamination in the water samples [56]. This approach highlighted the role of cloud technologies in bridging gaps between scientific disciplines such as biology and computer science.

### Electrophysiology devices

The transfer of electrical signals between cells is a fundamental concept in animal physiology, heart function, and neuronal communication. Several pedagogical tools have been developed to facilitate the learning of electrophysiology. For example, the SpikerBox is a device designed for measuring electrical signals in insects [57,58]. This tool can be paired with a cellphone for experiments where students use sewing pins to connect to the legs of invertebrates. This equipment has been instrumental in teaching electrophysiology principles using organisms such as cockroaches [8,57,58], crickets [59], grasshoppers [60], and mantis shrimps [61]. Further modifications allowed the recording of electrical activity in human muscles and in plants [8,62]. Amid the COVID-19 pandemic, SpikerBox-based experiments were adapted for remote execution, with teachers sending kits to the students and having them perform experiments at home [63]. Although innovative, these experiments have been hampered by the logistics necessary to deliver instruments to each student rather than having remote connectivity.

RoboRoach is a system that implants electrodes into the antennas of cockroaches and enables their remote control through Bluetooth [64]. This system allows students to manipulate cockroach movements through a cellphone app that delivers microstimulation to the electrodes [64]. Coupling steady-state visual evoked potential (SSVEP)-based electroencephalography (EEG) with the RoboRoach has been used to control cockroach behavior with the human brain, effectively creating a brain-to-brain interface between humans and cockroaches [65]. The RoboRoach has been used in a variety of courses in Bolivia, Mexico, Spain, and the USA [8,33,34].

However, electrophysiological recordings are relevant not only in whole organisms but also in cells, such as cardiomyocytes and neurons, growing in culture. Such techniques do more than verify the functional viability of the neurons: they also provide information about the complex patterns of communication and network dynamics inherent to neural assemblies. Educational modules have benefited from the use of multielectrode arrays (MEAs) that detect extracellular electrical signals generated by neurons [66,67]. Because MEAs are not invasive, they allow longitudinal tracking of the development and adaptive changes within neural networks over extended periods [68]. Furthermore, the ability to longitudinally track neural network evolution allows open- and closed-loop manipulations of these systems [69]. MEAs have been adapted to the cloud through a series of innovations. For example, the PiPhys system is a cloud-enabled device that uses a Raspberry Pi computer with a bioamplifier chip to facilitate voltage sampling [70]. This setup is compatible with several electrode probes, including rigid 2D and flexible MEAs, silicon probes, and tetrodes. The system uses MQTT (message queuing telemetry transport) for messaging across networked devices, complemented by data streaming to Amazon Web Services (AWS) for storage [70,71].

Commercial systems such as MaxOne (Maxwell Biosystems) have been adapted to the cloud for use in education [32]. MaxOne is a high-density MEA that has 26 000 electrodes in a single well. On average, each neuron in contact with MaxOne is covered by 3–4 electrodes, enabling precise spike sorting and network analysis. MaxOne has been used remotely in the classroom in combination with brain organoids to introduce mathematics and computer science concepts into neuroscience and stem cell biology [32]. Through this approach, the students were able to design custom stimulation patterns in neuronal tissue and assess their effect on neural plasticity and circuit behavior [32]. This approach was shown to develop the interest of students from non-biomedical degrees in further training in neuroscience and regenerative biology [32].

## Biological tools for live-cell biotechnology

An advantage of cloud technologies is that they enable students to work with complex models, including potentially pathogenic organisms and biohazardous materials that require biosafety level 2 or 3 environments, which are inaccessible to most schools around the world. The selection of the proper model organism is therefore no longer limited by specialized training or biosafety measures. Classrooms have leveraged several models, ranging from microorganisms and cell cultures to whole organisms. We review some examples in the following sections.

### Microorganisms

To date, bacteria have been the most common organisms used in cloud-enabled biotechnology education owing to their rapid growth and low maintenance costs. For example, *Escherichia coli* has been widely used in LoC systems to test for contamination in water samples and create context-aware educational modules [56,72]. Bacteria have also been labeled either with dyes [47] or through genetic engineering [20] to serve as ‘bioink’. Combining these bacteria with cloud-enabled liquid-handling robots has enabled students to print artistic renderings in Petri dishes and engage students without a biology background [47].

Other unicellular organisms have been of interest to the education community. *E. gracilis* has been often coupled to microscopy experiments owing to its phototactic behavior [18,19]. By enabling users to visualize these algae through a microscope and control the function of a light source remotely, educators have been able to introduce students to the scientific method and quantitative aspects of biology. This approach has been used to test the scalability of cloud technologies in the classroom, enabling >2300 remote experiments in 46 countries [19].

Protists, such as the slime mold, have been used as models for multicellular assembly [17]. This model has been used to supplement a theoretical graduate-level course taught to engineering and applied physics students [17]. This work served as a proof of principle for the integration of mathematics concepts using live-cell biotechnology and provided strong evidence for the preference of students for remote experiments over computer simulations [17].

### Mammalian tissue culture

Compared to microorganisms, maintaining mammalian tissue cultures is more challenging and costly [73]. Consequently, most undergraduate biology and biotechnology programs worldwide lack formal training in mammalian tissue culture [32]. Some in-person courses use primary cell cultures [74–77] or established cell lines [78–81] to teach basic tissue culture techniques. However, these courses often prioritize technical skills over enabling students to design complex experiments and make novel scientific discoveries.

Internet-connected microscopes have been valuable for creating open-ended educational modules that integrate cell culture models, such as neuroblastoma cells, into biology courses in Salinas, California [16]. This approach enabled students to test the effects of various drugs on neuroblastoma differentiation and survival [16]. Through this method, students were introduced to key concepts in cell signaling, stem cell identity, and fate acquisition, all of which are tested in standard biology exams.

Incorporating pluripotent stem cell (PSC) models into classroom settings has the potential to revolutionize undergraduate and medical training worldwide. PSCs hold significant promise in the medical field because patient-derived PSCs can provide insights into disease emergence, progression, and treatment [82,83]. Although biotechnology students often learn about the theory behind PSCs, hands-on experience with PSCs in the classroom is rare. Currently, most undergraduate students only work with PSCs in extracurricular research activities at select elite universities [32,35]. Recent technological advances have facilitated the integration of PSC-derived models, and successful testing has been conducted in community college and universities courses in Northern California.

PSC-derived organoids are particularly attractive in the pharmaceutical industry [41]. However, hands-on training in the generation and maintenance of organoids remains mostly confined to research laboratories, and their use in the in-person classroom has only started recently [84,85]. Using cloud-connected microscopes, undergraduate students have been able to assess the effects of different drugs on organoid growth [32]. Furthermore, the use of cloud-connected MEAs enabled mathematics students to design stimulation patterns to study neural plasticity [32]. Interestingly, surveying both groups of students led to similar conclusions: the students acknowledged that performing cloud experiments enabled them to conduct experiments that would not normally be available to them. They all reported increased interest in the topic and greater desire to pursue careers in stem cell research [32].

However, several key innovations will be necessary to expand the use of PSC-derived models in education. Implementing protocols to accelerate the production speed of the target cells while ensuring their homogeneity will facilitate the development of more complex educational modules. One promising approach is the overexpression of transcription factors (TFs) to manipulate stem cell fate [86]. For instance, NGN2 overexpression in PSCs triggers the rapid induction of neuronal genes [87]. Supplementing TFs with small molecules can modulate additional pathways for fate refinement. This strategy has successfully induced dopaminergic neurons [88] and glutamatergic neurons [89]. The introduction of previously untested small molecules will give students the opportunity to engage in the discovery process.

### Genetically engineered cell lines

The use of genetically engineered tools has been a pivotal aspect of biotechnology education. The first use of green fluorescent protein (GFP) in the classroom dates back to the Protein Purification: Isolation, Analysis, and Characterization of GFP course at Rutgers University in 1989 [90]. Since then GFP has been used in a variety of courses ranging from cloning and protein isolation to tracking individual cells in small animals [90,91]. Indeed, genetic reporters have become favored tools to teach concepts in chemistry, genetics, genome engineering, and bioethics [90,92].

To date, all cloud-enabled microscopy-based experiments in education have been conducted using brightfield imaging [16,18,19,32]. However, several inexpensive microscopes capable of fluorescent imaging could be adapted to the cloud [93–95]. The development of internet-controlled fluorescent microscopes will democratize the use of genetic fluorophore reporters in the classroom. For example, genetic reporters could be used to perform highly complex experiments using mammalian cells, such as visualizing morphogens [96]. The use of calcium indicators [97,98] could enable students to create models of networks in neural cultures. Furthermore, the use of neurochemical-sensing G-protein-coupled receptor activation-based (GRAB) sensors [99] could facilitate multimodal data analysis and modeling in graduate-level courses.

Finally, the education field can benefit from the use of engineered cell lines with CRISPR-mediated activation/inhibition (CRISPRa/i) systems [100]. This approach can incorporate computational tools into the classroom and enable students to design guide RNAs for testing [101]. Following this, students can analyze the impacts of CRISPRa/i manipulation using microscopy and electrophysiology techniques. This strategy will empower students to perform genetic screens to study cell signaling and complement current modules in drug screens [16].

### Multicellular organisms

Cloud technologies have been integrated into whole organisms in various classroom contexts. For example, two separate groups utilized plants and cloud-connected equipment to engage students [102,103]. In Spain, the Spike system has been used in agronomic engineering courses to monitor environmental metrics such as carbon dioxide levels, light intensity, temperature, and soil moisture [102]. In Israel, high school students measured the same parameters in a smart greenhouse as an introduction to the scientific method and experimental design [103]. In both scenarios, the students demonstrated an increased understanding of the topic and high levels of comfort with the technologies [102,103].

Several small animals have been used with cloud microscopes to teach biological processes and investigate the toxicity of different reagents [16]. For example, planaria worms have been used to study photophobic behavior, and *Xenopus tropicalis* has been used to demonstrate the normal developmental process [36]. Zebrafish has been a preferred model organism to examine the toxic effects of fertilizer byproducts, chlorine dioxide, and graphene nanoparticles [16].

## Connecting the unconnected

The majority of systems have used their own interfaces for enabling interaction [16,18,102]. Alternatively, some systems have relied on commercial streaming platforms such as YouTube [32]. The use of these platforms benefits from adaptive streaming capabilities [104], allowing their use even in regions with low internet bandwidth. Because internet speed is a major impediment to remote education [105], this represents a significant advance in reducing inequalities.

Several software architectures have been proposed for integrating multiple cloud-enabled devices within a single system [71,106–108]. In these architectures, systems connect to services that enable user control. Data is stored in external servers, and a custom-made application allows users to interact with the devices. These architectures then facilitate data streaming from the devices using services such as Redis [71].

**Figure 2.**
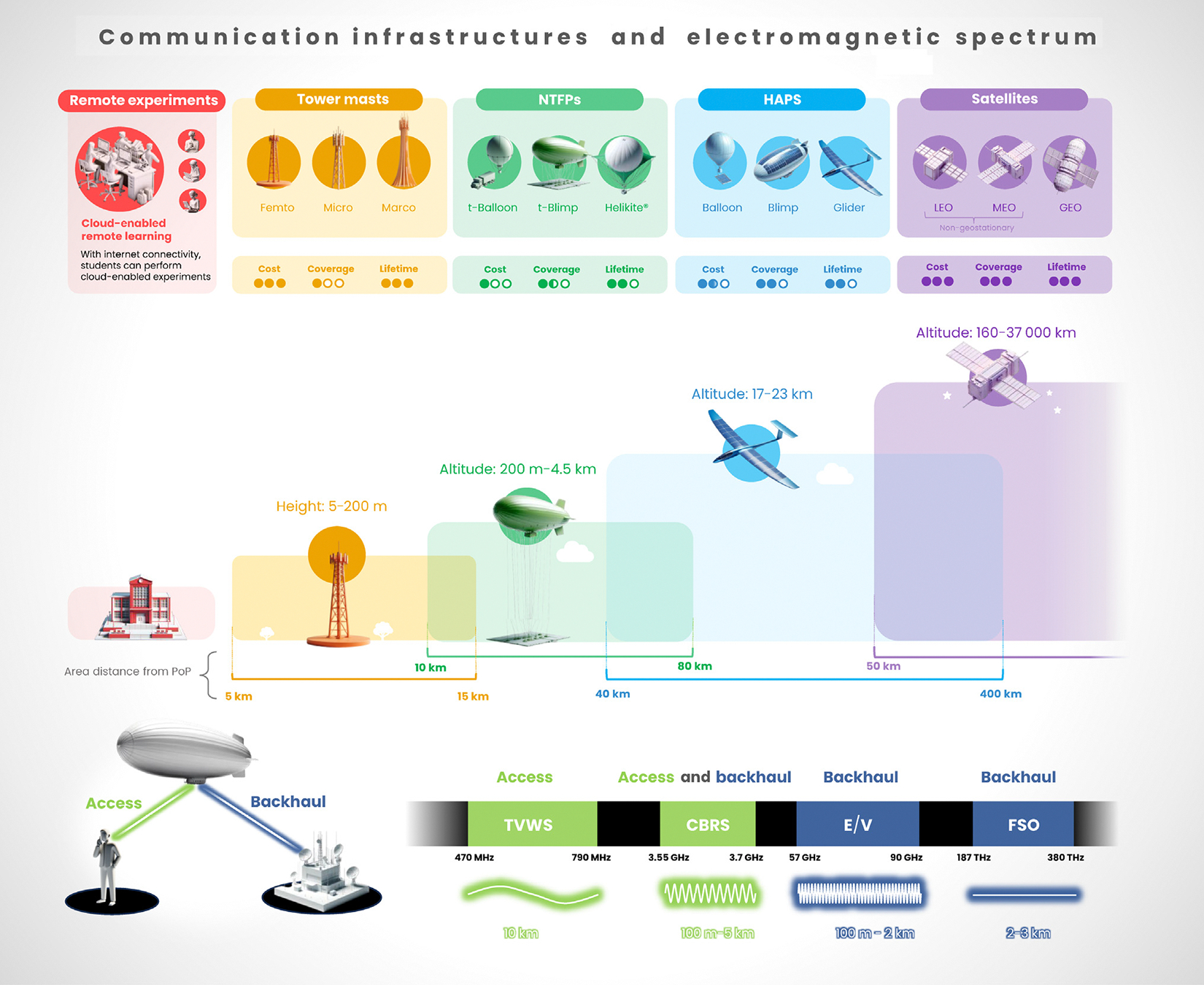
Solutions to bridge the digital divide. Cost-effective communication infrastructures and electromagnetic solutions. The communication infrastructures include tower masts, networked tethered flying platforms (NTFPs), high-altitude platform stations (HAPS), and satellites. The electromagnetic solutions encompass access solutions such as TV white space (TVWS), backhaul solutions including E/V bands and free space optics (FSO), and versatile options such as the citizens broadband radio service (CBRS) which serve as both access and backhaul solutions. Abbreviations: GEO, geostationary orbit; LEO, low Earth orbit; MEO, medium Earth orbit.

Students from under-represented groups often live in areas where internet connectivity is scarce or absent [109–111]. Solutions involve cost-effective communication infrastructures (Box 1) and electromagnetic spectrum options (Box 2) to enable mobile network operators to extend services to remote areas (Figure 2).

## Considerations when designing cloud-based courses

Effectively delivering a cloud-based live-cell biotechnology course requires consideration of several elements, including the length of the experiment and the teaching environment. For instance, outreach experiments in informal settings such as museums may benefit from using microorganisms and other rapidly responding biological systems such as optogenetically responsive cells. By contrast, university courses taught in formal settings may gain more from incorporating complex models such as organoids.

Cloud-based courses offer high customization. However, formal courses that leverage these technologies must introduce students to experiments in a stepwise manner. Although many approaches have been developed [16,20,31,56,112–114], the general design principles are summarized in Table 1.

Although cloud technologies can provide access to models that are typically unavailable in undergraduate labs, important regulatory and ethical considerations must be kept in mind during course design. For example, the use of complex organisms such as vertebrates requires Institutional Animal Care and Use Committee (IACUC) approval. Because IACUC standards can vary between institutions [115], it is crucial to understand the specific regulations applicable at the experimental site. Similarly, when using novel models such as human neurons and brain organoids, incorporating ethical lessons into the curriculum is beneficial.

A significant aspect of course design is obtaining approval from the Institutional Review Board (IRB). This is particularly important when data will be collected with the intention of publication because the majority of users, including students and minors, are considered to be susceptible populations. Traditional IRB applications may not be structured to address the uncertainties inherent in cloud-based courses, such as the number of users accessing the modules or the variability in user locations. Therefore, researchers should engage in open conversations with their IRBs and be prepared to modify their experimental designs as necessary.

Collaborative online international learning (COIL) is an effective approach for enabling students from different cultures to work together [12]. However, implementing COIL in practice can be challenging because it is often limited by language barriers and differences in academic calendars between geographically distant education systems [116]. In addition, training the teachers who interact directly with the students has been overlooked to date. Ensuring that teachers are familiar with the technologies is crucial for facilitating interactions [117]. We propose a multimodular course structure that can be adapted for teacher training across different cloud technologies (Table 2).

International outreach projects in developing regions often encounter resistance from local educators who may view these activities as neocolonial practices [8,35]. It is essential to balance enabling students to perform cutting-edge experiments with the development of projects relevant to the communities involved. Some approaches have used themes of mutual interest, such as COVID-19 treatment combined with COIL methodologies [16]. Other approaches may focus on indigenous plants or context-specific chemicals, such as fertilizers for agricultural communities [16].

## Bridging frugal science and virtual laboratories

Beyond cloud-based live-cell biotechnology, various educational approaches have been proposed, each with their own advantages and disadvantages (Table 3). Frugal science, for instance, involves the use of low-cost or repurposed equipment to perform laboratory functions [118]. This provides hands-on training but is often hindered by high shipment costs for equipment and reagents, as well as customs regulations concerning biological materials [119].

At the other end of the spectrum are virtual labs which use computer simulations to train students in basic techniques [85,120]. Although these simulations can create complex laboratory environments, they are often unaffordable by most schools and cannot accurately replicate the experience of scientific uncertainty and true discovery.

Importantly, frugal and virtual laboratories are often seen as alternatives but can be complementary to cloud-based education. For example, the HCI interface between users and cloud equipment could leverage virtual lab environments to enhance the user experience. Similarly, frugal approaches could serve as introductory activities to familiarize students with concepts that will be explored in more complex cloud-based experiments.

## Conclusion and future perspectives

Despite being in early stages, cloud-enabled live-cell biotechnology has been shown to be effective in enabling scientific inquiry and discovery in students at multiple educational levels. The combination of these technologies with pedagogical innovations, such as project-based learning (PBL; Box 3), has led to improved STEM identity and knowledge comparable to that provided by in-person hands-on courses among students from under-represented backgrounds in the developing world [16]. An important lesson learned from this work is that under-represented students were particularly interested in pursuing careers after conducting PBL-based experiments that related to issues relevant to their own community [16,56], highlighting the importance of personalizing educational material.

Biotechnology experiments using the cloud have been performed in four primary locations: Northern California; Cambridge, MA; Madrid, Spain; and Haifa, Israel. However, students conducting these experiments have been located >50 countries worldwide. This approach implies that a few experimental ‘hubs’ can theoretically serve students in virtually every region of the world, and could thus achieve the SDG4 mandate to ensure inclusive and equitable quality education and promote lifelong learning opportunities for all. This marks an important departure from other approaches, such as the major push by the United Nations Development Program which has established 91 ‘Accelerator Labs’ globally [121].

The move towards using cloud technologies has the potential to save millions of dollars of investment in laboratory infrastructure and operation [10,35]. However, it is important to note that thus far the vast majority of remote students have been located in high- and upper middle-income countries, while students in lower-income countries have been left behind (Box 3). This is partly due to the lack of infrastructure, including internet delivery, which would be crucial in bridging the digital divide between regions of the world.

An advantage of cloud technologies is that students can collaborate despite being located in physically distant regions of the world. This opens up the possibility of conducting comparative educational projects to understand the impact of different interventions on the students and communities involved. However, there is still a crucial need to create educational rubrics and tools to better measure the impact of educational projects [122–124], which, in turn, will enable improvements in the curricula.

In summary, there is a growing momentum to use novel engineering, software, biological tools, and pedagogical methods to deliver live-cell biotechnology education to students across the world. This combination has the potential to create lasting impacts on society and reduce educational inequalities in a scalable and sustainable manner (see Outstanding questions).

## Figures and Tables

**Table 1.** Course design for cloud-enabled live-cell biotechnology courses

Module	Steps to take
Module 1. Introduction	Begin with an introduction that can take the form of a passive observation, a lecture, or a tutorial on operating the equipment. The aim is to familiarize students with the technology and the biological phenomena they will study.
Module2. Experimentation	Engage students in experimentation, which can be either interactive (preferred) or scripted (suitable for low-bandwidth scenarios). In the interactive mode, students explore biological phenomena freely. In the scripted mode, students follow a prewritten set of commands executed by the cloud-enabled equipment.
Module 3. Qualitative data analysis	Guide students through qualitative data analysis to help them to focus on specific phenomena. This step primes them for more detailed study and observation.
Module 4. Hypothesis generation	Encourage students to generate hypotheses that can be tested quantitatively. Examples include studying the behavior of cells and organisms or the effects of drugs on tissue.
Module 5 (optional). Introduction to analysis software	If needed, introduce students to analysis software, such as ImageJ, depending on the hypotheses they generate. This helps them to analyze their data effectively.
Module 6. Quantitative data analysis	Task students with performing quantitative analysis ofthe data. Examples include measuring cell migration distances, rates of mitosis or apoptosis, and morphologies induced by cell differentiation.
Module 7 (optional). Self-guided explorations	Enable students to undertake self-guided experiments, reanalyze their data with new hypotheses, or analyze data from their peers in several cloud-based courses.
Module 8. Summary and reflections	Conclude with a summary and reflection session in which students validate or negate their hypotheses and share their findings. This can include course discussions, posters, presentations, or creating YouTube videos for the general public.

**Table 2.** Suggested syllabus for training teachers in cloud-based live-cell biotechnology

Module	Topics to be covered
Module 1. Introduction to cloud laboratories	Overview of cloud labs and their importance in modern science education
	Discussion of the benefits of using cloud labs for teaching biotechnology and other sciences
Module 2. Exploring cloud lab platforms	Introduction to cloud lab platforms (e.g., microscopy, electrophysiology, liquid-handling robots, lab-on-a-chip)
	Demonstration of navigating the available interface and accessing experiments
	Guided exploration of sample experiments
Module 3. Integrating cloud labs into the curriculum	Strategies for incorporating cloud labs into the existing curriculum
	Discussion of aligning cloud lab activities with learning objectives and standards
Module 4. Q&A and wrap-up	Open forum for questions, discussion, and sharing of experiences
	Recap of key takeaways and resources for further exploration

**Table 3.** Alternatives to cloud-based live-cell biotechnology

	Frugal science	Cloud-based live-cell biotechnology	Virtual labs
Biotechnology equipment costs	Low-cost equipment	Can be either low-cost or professional equipment	Not necessary
Computer equipment	Not necessary	Standard computers	May require computers with high random access memory (RAM)
Reagents	Yes, although usually low-cost	Yes	Not necessary
Reagents and equipment shipment	Can be costly for international shipments	Not necessary	Not necessary
Enable complex experiments	Usually only simple experiments	Yes	Yes
Enable true scientific inquiry	Yes	Yes	No, because of predesigned modules
Enable collaborative online learning	Usually no	Yes	Usually no
Enable context-aware teaching	Yes	Yes	Usually no, because of predesigned modules

## Highlights

Cloud-enabled live-cell biotechnology offers students the opportunity to participate in real-time experiments using their own devices.

Advances in engineering internet-connected wet laboratory equipment to create new biological models, and the use of open-source software architectures to integrate multiple devices, have all contributed to the development of cloud-enabled live-cell biotechnology.

In addition, methods to extend internet access to remote areas and improvements in pedagogical approaches for remote experimental education are crucial for expanding the reach of biotechnology education.

Live-cell biotechnology holds promise for positively impacting on communities worldwide and in contributing to the achievement of the United Nations Sustainable Development Goals.

## Box 1. Communication Infrastructures

The effectiveness of various communication infrastructures and solutions leveraging the electromagnetic spectrum largely depends on the proximity of an area to an optical fiber point of presence (PoP). A PoP is a hub or access point where different networks or communication lines come together. The closer a remote area is to a PoP, the easier and more cost-effective it is to extend high-quality internet services to that area.

Satellites (areas located >50 km from a PoP)

For internet gateways situated close to an optical fiber PoP, very small aperture terminals (VSATs) within these remote areas should be cost-effective and can potentially be powered by renewable energy sources such as solar panels or wind turbines [125,126]. Geostationary satellites, that are fixed relative to the Earth, are ideal for broadcasting, whereas non-geostationary satellites, that orbit closer to the Earth, provide broadband internet with reduced signal latency [127,128].

High-altitude platform stations (HAPS; for areas located 40–400 km from a PoP)

HAPS are advanced aerial technologies operating within the stratosphere at altitudes ranging from 17 km to 23 km [129,130]. These include fixed-wing gliders, blimps, and balloons, are powered by solar energy, and can remain airborne for months [131]. They offer extensive communication coverage compared to traditional terrestrial communication infrastructure. HAPS are also cost-effective and rapidly deployable on site. They are suitable in areas where terrestrial infrastructure is limited or non-existent [132].

Networked tethered flying platforms (NTFPs; areas located 10–80 km from a PoP)

NTFPs include blimps, balloons, and drones that are anchored to the ground via a tether that supplies continuous power and internet connectivity [133]. NTFPs offer a blend of ease of deployment and cost-effectiveness, making them an attractive option for sustained aerial operations. They are designed to remain airborne for extended periods (up to 1 month) before needing to be reeled in for maintenance or refueling, after which they can be launched again [134,135].

Tower masts (areas located 5–15 km from a PoP)

Tower masts, which are the traditional terrestrial communication infrastructure, range in height from 5 m to 100 m. Their internet coverage is influenced by the height of the mast and the surrounding terrain. The masts are strategically placed to maximize internet coverage and connectivity in remote areas [136].

## Box 2. The electromagnetic spectrum

The electromagnetic spectrum is a finite resource in terms of the availability of specific frequency bands for various applications, leading to high costs in the development of digital and telecommunications infrastructure. Consequently, strategies such as the use of licensed frequencies and infrastructure sharing among mobile network operators can optimize resource utilization. In addition, exploring the unlicensed (free-to-use) spectrum for access and backhaul segments offers a cost-effective alternative. ‘Access’ is the segment that connects users to their service provider, and acts as the ‘last mile’ for internet connectivity through WiFi, mobile networks (4G/5G), or broadband internet. It enables activities such as browsing and streaming. ‘Backhaul’ refers to the infrastructure that links the access network to the internet core, thereby functioning as a data highway. It can transport vast amounts of data, often via optical fiber or wireless technologies, thus ensuring a swift and stable internet connection. The different frequency segments are listed in the following sections.

TV white space (TVWS; 470–790 MHz)

TVWS comprises underutilized frequencies located between TV channels, and offers a unique capability for wireless communication in rural and underserved areas. With the ability to cover long distances (typically 10 km or more) and penetrate obstacles, TVWS is predominantly used for access networks that provide connectivity in areas lacking traditional infrastructure [137].

Citizens broadband radio service (CBRS; 3.55–3.7 GHz)

CBRS is a regulatory framework that is designed to allow shared wireless access within a specific portion of the spectrum. CBRS enables both licensed and unlicensed users to coexist, offering a flexible and efficient use of the spectrum. Given its coverage range from a few hundred meters to several km, CBRS is versatile, serving both as an access solution in urban and rural settings and as a backhaul option, particularly in densely populated areas [138].

E/V band (57–90 GHz)

This high-frequency band is tailored for point-to-point microwave links that can provide high-speed internet over a short-distance. Owing to its range – from a few hundred meters to ~2 km – and the necessity for line-of-sight, the E/V band is particularly suitable for backhaul applications and enables robust connections between network nodes or within mobile networks and broadband services [139].

Optical spectrum free space optics (FSO; 187–380 THz)

FSO uses light beams to transmit data through the air [140]. FSO offers a high-speed alternative to traditional radio frequency communications, with the advantage of being highly secure and immune to interference. It is particularly useful for creating quick-to-deploy, point-to-point links between buildings or across short distances where laying fiber is impractical [141]. Although atmospheric conditions such as fog and rain can affect its range (up to 2–3 km is typically achievable under clear weather conditions), the high throughput of FSO makes it an excellent choice for backhaul applications in urban environments [142].

## Box 3. Pedagogical innovations: bringing technologies to the classroom

Over recent years cloud-enabled live-cell biotechnology experiments have evolved from simple, predetermined projects [18] to dynamic experiments that incorporate scientific uncertainty and true discovery [16,20,32]. These experiments effectively integrate project-based learning (PBL), a pedagogical approach that starts with real-world problems and promotes inquiry-based learning. This student-centered method encourages learners to actively engage in their educational journey. This approach has been particularly beneficial for students from under-represented backgrounds, leading to an increase in STEM identity and knowledge acquisition [8,143]. Similarly to in-person PBL teaching [8], students have reported an increased level of STEM identity and knowledge gained in the topic [16,102,103]. Importantly, many students reported they see PBL-based education as ‘different’ and ‘exciting’ compared to other remote teaching techniques [16,56].

Large-scale education initiatives have demonstrated that access to technology alone does not sufficiently enhance learning among underserved populations. The one laptop per child (OLPC) project, for instance, distributed over 3 million low-cost laptops to students in developing countries but often resulted in decreased academic performance and no significant improvement in cognitive skills [144,145]. Similarly, analyses of MOOCs such as Coursera and EdX indicate that these resources typically benefit students from more advantaged backgrounds [146]. Therefore, although new technologies can lead to innovative learning resources, much care needs to be taken to couple them with optimal pedagogical techniques.

Cloud-based live-cell biotechnology education tools have already been deployed in >50 countries (Figure I). A comprehensive set of collaboration tools and diverse resources, aligned with well-established standards in educational systems, has become crucial for effectively integrating cloud technologies into the educational sector.

## Outstanding questions

What are the limitations to cloud-enabled live-cell biotechnology in the classroom? What equipment and data-generation modalities remain to be explored?

Can algorithms be trained to assist in teaching students? For example, would pairing artificial intelligence with live-cell biotechnology enable computer predictions that could inform students to modify their experiments? Would this approach result in novel scientific discoveries?

How would we integrate the data generated by the different data modalities in a manner that makes multimodal files easily shareable and adaptable to open-source software?

What new metrics should be explored to measure the impact of cloud-enabled live-cell biotechnology interventions in different communities?

## References

[1] Boeren E (2019) Understanding Sustainable Development Goal (SDG) 4 on ‘quality education’ from micro, meso and macro perspectives. Int. Rev. Educ. 65, 277–294

[2] Heleta S and Bagus T (2021) Sustainable development goals and higher education: leaving many behind. High. Educ. 81, 163–177

[3] Ferreira-Meyers K and Dhakulkar A (2021) Can open science offer solutions to science education in Africa? In Radical Solutions for Education in Africa: Open Education and Self-directed Learning in the Continent (Burgos D and Olivier J, eds), pp. 149–174, Springer

[4] Veidemane A (2021) Inclusive higher education access for underrepresented groups: it matters, but how can universities measure it? Soc. Incl. 9, 44–57

[5] Barber K and Mostajo-Radji MA (2020) Youth networks’ advances toward the sustainable development goals during the COVID-19 pandemic. Front. Sociol. 5, 589539 33869518 10.3389/fsoc.2020.589539 PMC8022559

[6] Maryanti R (2022) Sustainable development goals (SDGs) in science education: definition, literature review, and bibliometric analysis. J. Eng. Sci. Technol. 17, 161–181

[7] Wibowo YG and Sadikin A (2019) Biology in the 21st-century: transformation in biology science and education in supporting the sustainable development goals. J. Pendidikan Biol. Indones. 5, 285–296

[8] Ferreira LMR (2019) Effective participatory science education in a diverse Latin American population. Palgrave Commun. 5, 63

[9] Kidman G (2010) What is an ‘interesting curriculum’ for biotechnology education? students and teachers opposing views. Res. Sci. Educ. 40, 353–373

[10] Mostajo-Radji MA (2023) A Latin American perspective on neurodiplomacy. Front. Med. Technol. 4, 1005043 36712171 10.3389/fmedt.2022.1005043 PMC9880232

[11] Armbrust M (2010) A view of cloud computing. Commun. ACM 53, 50–58

[12] Rubin J (2017) Embedding collaborative online international learning (COIL) at higher education institutions. Intern. High. Educ. 2, 27–44

[13] Rihm SD (2024) Transforming research laboratories with connected digital twins. Nexus 1, 100004

[14] Amselem S (2019) Remote controlled autonomous microgravity lab platforms for drug research in space. Pharm. Res. 36, 183 31741058 10.1007/s11095-019-2703-7

[15] Koch C (2022) Next-generation brain observatories. Neuron 110, 3661–3666 36240770 10.1016/j.neuron.2022.09.033

[16] Baudin PV (2022) Cloud-controlled microscopy enables remote project-based biology education in underserved Latinx communities. Heliyon 8, e11596 36439758 10.1016/j.heliyon.2022.e11596 PMC9681640

[17] Hossain Z (2015) Interactive cloud experimentation for biology: an online education case study. In Proceedings of the 33rd Annual ACM Conference on Human Factors in Computing Systems, pp. 3681–3690, ACM

[18] Hossain Z (2016) Interactive and scalable biology cloud experimentation for scientific inquiry and education. Nat. Biotechnol. 34, 1293–1298 27926727 10.1038/nbt.3747

[19] Hossain Z (2017) Authentic science inquiry learning at scale enabled by an interactive biology cloud experimentation lab. In L@S2017: Proceedings of the 4th (2017) ACM Conference on Learning at Scale, pp. 237–240, ACM

[20] Perry E (2022) How to grow (almost) anything: a hybrid distance learning model for global laboratory-based synthetic biology education. Nat. Biotechnol. 40, 1874–1879 36510008 10.1038/s41587-022-01601-x

[21] Jackson LA (2006) Does home internet use influence the academic performance of low-income children? Dev. Psychol. 42, 429 16756435 10.1037/0012-1649.42.3.429

[22] del Portillo I (2021) Connecting the other half: Exploring options for the 50% of the population unconnected to the internet. Telecommun. Policy 45, 102092

[23] Pattnaik J (2023) Challenges to remote instruction during the pandemic: a qualitative study with primary grade teachers in India. Early Childhood Educ. J. 51, 675–684 35287284 10.1007/s10643-022-01331-4 PMC8907896

[24] Schmidt D and Power SA (2021) Offline world: the internet as social infrastructure among the unconnected in quasi-rural Illinois. Integr. Psychol. Behav. Sci. 55, 371–385 32827073 10.1007/s12124-020-09574-9 PMC7442287

[25] Powell A (2010) The essential Internet: digital exclusion in low-income American communities. Policy Internet 2, 161–192

[26] Ochoa RG (2022) Mobile internet adoption in West Africa. Technol. Soc. 68, 101845

[27] Datta A (2018) Bridging the digital divide: challenges in opening the digital world to the elderly, poor, and digitally illiterate. IEEE Consum. Electr. Mag. 8, 78–81

[28] Warf B (2011) Geographies of global Internet censorship. GeoJournal 76, 1–23

[29] Reisdorf BC and DeCook JR (2022) Locked up and left out: formerly incarcerated people in the context of digital inclusion. New Media Soc. 24, 478–495

[30] Győrödi C (2015) A comparative study: MongoDB vs. MySQL. In 2015 13th International Conference on Engineering of Modern Electric Systems (EMES), pp. 1–6, IEEE

[31] Hossain Z (2018) Design guidelines and empirical case study for scaling authentic inquiry-based science learning via open online courses and interactive biology cloud labs. Int. J. Artif. Intell. Educ. 28, 478–507

[32] Elliott MAT (2023) Internet-connected cortical organoids for project-based stem cell and neuroscience education. eNeuro 10, ENEURO.0308–23.2023 10.1523/ENEURO.0308-23.2023 PMC10755643 38016807

[33] Carosso GA (2019) Developing brains, developing nations: can scientists be effective non-state diplomats? Front. Educ. 4, 95

[34] Carosso GA (2019) Scientists as non-state actors of public diplomacy. Nat. Hum. Behav. 3, 1129–1130 31427786 10.1038/s41562-019-0716-1

[35] Mostajo-Radji MA (2022) The emergence of neurodiplomacy. iScience 25, 104370 35601914 10.1016/j.isci.2022.104370 PMC9120262

[36] Ly VT (2021) Picroscope: low-cost system for simultaneous longitudinal biological imaging. Commun. Biol. 4, 1261 34737378 10.1038/s42003-021-02779-7 PMC8569150

[37] Baudin PV (2022) Low cost cloud based remote microscopy for biological sciences. Internet Things 18, 100454

[38] Awandare G (2020) Science advisers around the world on 2020. Nature 588, 586–588 33340028 10.1038/d41586-020-03557-x

[39] Mostajo-Radji MA (2021) Pseudoscience in the times of crisis: How and why chlorine dioxide consumption became popular in Latin America during the COVID-19 pandemic. Front. Polit. Sci. 3, 621370

[40] Carozza SE (2008) Risk of childhood cancers associated with residence in agriculturally intense areas in the United States. Environ. Health Perspect. 116, 559–565 18414643 10.1289/ehp.9967 PMC2290991

[41] Salick MR (2021) The future of cerebral organoids in drug discovery. Semin. Cell Dev. Biol. 111, 67–73 32654970 10.1016/j.semcdb.2020.05.024

[42] Boulter E (2022) The LEGO^®^ brick road to open science and biotechnology. Trends Biotechnol. 40, 1073–1087 35314074 10.1016/j.tibtech.2022.02.003

[43] Faiña A (2020) EvoBot: an open-source, modular, liquid handling robot for scientific experiments. Appl. Sci. 10, 814

[44] Gerber LC (2017) Liquid-handling Lego robots and experiments for STEM education and research. PLoS Biol. 15, e2001413 28323828 10.1371/journal.pbio.2001413 PMC5360201

[45] Faiña A (2016) EvoBot: an open-source, modular liquid handling robot for nurturing microbial fuel cells. In ALIFE 2016, Fifteenth International Conference on the Synthesis and Simulation of Living Systems, pp. 626–633, MIT Press Direct

[46] Nejatimoharrami F (2017) New capabilities of EvoBot: a modular, open-source liquid-handling robot. SLAS Technol. 22, 500–506 28378607 10.1177/2472630316689285

[47] Gome G (2019) OpenLH: open liquid-handling system for creative experimentation with biology. In Proceedings of the Thirteenth International Conference on Tangible, Embedded, and Embodied Interaction, pp. 55–64, ACM

[48] Arshavsky-Graham S and Segal E (2022) Lab-on-a-chip devices for point-of-care medical diagnostics. In Microfluidics in Biotechnology (Bahnemann J and Grünberger A, eds), pp. 247–265, Springer International 10.1007/10_2020_127 32435872

[49] Sano T (2022) All-in-one optofluidic chip for molecular biosensing assays. Biosensors 12, 501 35884304 10.3390/bios12070501 PMC9313335

[50] Pol R (2017) Microfluidic lab-on-a-chip platforms for environmental monitoring. TrAC Trends Anal. Chem. 95, 62–68

[51] Li D (2014) Single-phase electrokinetic flow in microchannels. In Heat Transfer and Fluid Flow in Minichannels and Microchannels (2nd edn) (Kandlikar SG , eds), pp. 175–219, Butterworth–Heinemann

[52] Bridle H (2016) Design of problem-based learning activities in the field of microfluidics for 12- to 13-year-old participants – small plumbing!: empowering the next generation of microfluidic engineers. Microfluid. Nanofluid. 20, 103

[53] Fintschenko Y (2011) Education: a modular approach to microfluidics in the teaching laboratory. Lab Chip 11, 3394–3400 21909517 10.1039/c1lc90069b

[54] Rackus DG (2019) ‘Learning on a chip’: microfluidics for formal and informal science education. Biomicrofluidics 13, 041501 31431815 10.1063/1.5096030 PMC6697029

[55] Wietsma JJ (2018) Lab-on-a-chip: frontier science in the classroom. J. Chem. Educ. 95, 267–275 30258250 10.1021/acs.jchemed.7b00506 PMC6150665

[56] Sano T (2024) Internet-enabled lab-on-a-chip technology for education. Sci. Rep. 14, 14364 38906940 10.1038/s41598-024-65346-0 PMC11192768

[57] Marzullo TC and Gage GJ (2012) The SpikerBox: a low cost, open-source bioamplifier for increasing public participation in neuroscience inquiry. PLoS One 7, e30837 22470415 10.1371/journal.pone.0030837 PMC3310049

[58] Prabhakaran G and Voit W (2014) Using Spikerbox as an education toolkit of body sensor network for brain activity monitoring. In The 11th Body Sensor Networks Conference, pp. 1–2, IEEE

[59] Dagda RK (2013) Using Crickets to Introduce Neurophysiology to Early Undergraduate Students. J. Undergrad. Neurosci. Educ. 12, A66–A74 24319394 PMC3852874

[60] Nguyen DMT (2017) Grasshopper DCMD: an undergraduate electrophysiology lab for investigating single-unit responses to behaviorally-relevant stimuli. J. Undergrad. Neurosci. Educ. 15, A162–A173 28690439 PMC5480846

[61] Pollak DJ (2019) An electrophysiological investigation of power-amplification in the ballistic mantis shrimp punch. J. Undergrad. Neurosci. Educ. 17, T12–T18 31360136 PMC6650263

[62] Oezkaya B and Gloor PA (2020) Recognizing individuals and their emotions using plants as bio-sensors through electrostatic discharge. ArXiv, Published online May 10, 2020. 10.48550/arXiv.2005.04591

[63] Hanzlick-Burton C (2020) Developing and implementing low-cost remote laboratories for undergraduate biology and neuroscience courses. J. Undergrad. Neurosci. Educ. 19, A118–A123 33880099 PMC8040849

[64] Stojnić A (2017) Only cyborgs and cockroaches. Perform. Res. 22, 123–128

[65] Li G and Zhang D (2017) Brain–computer interface controlling cyborg: a functional brain-to-brain interface between human and cockroach. In Brain–Computer Interface Research: A State-of-the-Art Summary (5) (Guger C , eds), pp. 71–79, Springer International

[66] Huang Y-T (2017) Positive feedback and synchronized bursts in neuronal cultures. PLoS ONE 12, e0187276 29091966 10.1371/journal.pone.0187276 PMC5665536

[67] Obien MEJ (2015) Revealing neuronal function through microelectrode array recordings. Front. Neurosci. 8, 423 25610364 10.3389/fnins.2014.00423 PMC4285113

[68] Negri J (2020) Assessment of spontaneous neuronal activity in vitro using multi-well multi-electrode arrays: implications for assay development. eNeuro 7, ENEURO.0080–19.2019 10.1523/ENEURO.0080-19.2019 PMC6984810 31896559

[69] Siegle JH (2017) Open Ephys: an open-source, plugin-based platform for multichannel electrophysiology. J. Neural Eng. 14, 045003 28169219 10.1088/1741-2552/aa5eea

[70] Voitiuk K (2021) Light-weight electrophysiology hardware and software platform for cloud-based neural recording experiments. J. Neural Eng. 18, 066004 10.1088/1741-2552/ac310a PMC8667733 34666315

[71] Parks DF (2022) IoT cloud laboratory: internet of things architecture for cellular biology. Internet Things 20, 100618 10.1016/j.iot.2022.100618 PMC10305744 37383277

[72] Golberg A (2014) Cloud-enabled microscopy and droplet microfluidic platform for specific detection of *Escherichia coli* in water. PLoS ONE 9, e86341 24475107 10.1371/journal.pone.0086341 PMC3903517

[73] Grineski S (2018) The conundrum of social class: disparities in publishing among STEM students in undergraduate research programs at a Hispanic majority institution. Sci. Educ. 102, 283–303 30416213 10.1002/sce.21330 PMC6224159

[74] Burdo JR (2013) Using chick forebrain neurons to model neurodegeneration and protection in an undergraduate neuroscience laboratory course. J. Undergrad. Neurosci. Educ. 11, A178–A186 23805059 PMC3692248

[75] Catlin R (2016) Using cultured mammalian neurons to study cellular processes and neurodegeneration: a suite of undergraduate lab exercises. J. Undergrad. Neurosci. Educ. 14, A132–A137 27385922 PMC4917344

[76] Haskew-Layton RE and Minkler JR (2020) Chick embryonic primary astrocyte cultures provide an effective and scalable model for authentic research in a laboratory class. J. Undergrad. Neurosci. Educ. 18, A86–A92 32848516 PMC7438172

[77] Lemons ML (2012) Characterizing mystery cell lines: student-driven research projects in an undergraduate neuroscience laboratory course. J. Undergrad. Neurosci. Educ. 10, A96–A104 23504583 PMC3598092

[78] Bowey-Dellinger K (2017) Introducing mammalian cell culture and cell viability techniques in the undergraduate biology laboratory. J. Microbiol. Biol. Educ. 18, 18.2.38 10.1128/jmbe.v18i2.1264 PMC5576768 28861134

[79] McIlrath V (2015) Using mouse mammary tumor cells to teach core biology concepts: a simple lab module. J. Vis. Exp. 100, e52528 10.3791/52528 PMC4544947 26132733

[80] Mozdziak PE (2004) An introductory undergraduate course covering animal cell culture techniques. Biochem. Mol. Biol. Educ. 32, 319–322 21706746 10.1002/bmb.2004.494032050381

[81] Phelan SA and Szabo E (2019) Undergraduate lab series using the K562 human leukemia cell line: model for cell growth, death, and differentiation in an advanced cell biology course. Biochem. Mol. Biol. Educ. 47, 263–271 30725506 10.1002/bmb.21222

[82] Ahani-Nahayati M (2021) Stem cell in neurodegenerative disorders; an emerging strategy. Int. J. Dev. Neurosci. 81, 291–311 33650716 10.1002/jdn.10101

[83] Azam S (2021) The ageing brain: molecular and cellular basis of neurodegeneration. Front. Cell Dev. Biol. 9, 683459 34485280 10.3389/fcell.2021.683459 PMC8414981

[84] Cvetkovic C (2024) Biofabrication of neural organoids: an experiential learning approach for instructional laboratories. Biomed. Eng. Educ. 4, 409–419

[85] Ly VT (2024) Gamifying cell culture training: the ‘Seru-Otchi’ experience for undergraduates. Heliyon 10, E30469 38737237 10.1016/j.heliyon.2024.e30469 PMC11088318

[86] Zhao Y (2023) Transcription factor-mediated programming of stem cell fate. Trends Cell Biol. 33, 621–624 37236901 10.1016/j.tcb.2023.05.004

[87] Zhang Y (2013) Rapid single-step induction of functional neurons from human pluripotent stem cells. Neuron 78, 785–798 23764284 10.1016/j.neuron.2013.05.029 PMC3751803

[88] Sheta R (2023) Optimized protocol for the generation of functional human induced-pluripotent-stem-cell-derived dopaminergic neurons. STAR Protoc. 4, 102486 37515763 10.1016/j.xpro.2023.102486 PMC10400954

[89] Gu J (2024) Generation of a stably transfected mouse embryonic stem cell line for inducible differentiation to excitatory neurons. Exp. Cell Res. 435, 113902 38145818 10.1016/j.yexcr.2023.113902

[90] Ward WW (2000) Green fluorescent protein in biotechnology education. Methods Enzymol. 305, 672–680 10812631 10.1016/s0076-6879(00)05518-x

[91] Bujanda C and Anderson N (2022) Teaching the central dogma through an inquiry-based project using GFP. Am. Biol. Teach. 84, 33–37

[92] Burnette JM and Wessler SR (2013) Transposing from the laboratory to the classroom to generate authentic research experiences for undergraduates. Genetics 193, 367–375 23172853 10.1534/genetics.112.147355 PMC3567729

[93] Hasan MM (2016) A low-cost digital microscope with real-time fluorescent imaging capability. PLoS ONE 11, e0167863 27977709 10.1371/journal.pone.0167863 PMC5158004

[94] Miller AR (2010) Portable, battery-operated, low-cost, bright field and fluorescence microscope. PLoS ONE 5, e11890 20694194 10.1371/journal.pone.0011890 PMC2915908

[95] Ryan J (2020) Building your own neuroscience equipment: a precision micromanipulator and an epi-fluorescence microscope for calcium imaging. J. Undergrad. Neurosci. Educ. 19, A134–A140 33880101 PMC8040841

[96] Schilling TF (2016) Visualizing retinoic acid morphogen gradients. Methods Cell Biol. 133, 139–163 27263412 10.1016/bs.mcb.2016.03.003 PMC4933793

[97] Nakai J (2001) A high signal-to-noise Ca^2+^ probe composed of a single green fluorescent protein. Nat. Biotechnol. 19, 137–141 11175727 10.1038/84397

[98] Paredes RM (2008) Chemical calcium indicators. Methods 46, 143–151 18929663 10.1016/j.ymeth.2008.09.025 PMC2666335

[99] Sun F (2020) Next-generation GRAB sensors for monitoring dopaminergic activity in vivo. Nat. Methods 17, 1156–1166 33087905 10.1038/s41592-020-00981-9 PMC7648260

[100] Shivram H (2021) Controlling and enhancing CRISPR systems. Nat. Chem. Biol. 17, 10–19 33328654 10.1038/s41589-020-00700-7 PMC8101458

[101] Lovato TL and Cripps RM (2024) CRISPR classroom activities and case studies. In Rigor and Reproducibility in Genetics and Genomics (Dluzen DF and Schmidt MHM, eds), pp. 453–471, Elsevier

[102] Tabuenca B (2023) Generating an environmental awareness system for learning using IoT technology. Internet Things 22, 100756

[103] Tsybulsky D and Sinai E (2022) IoT in project-based biology learning: students’ experiences and skill development. J. Sci. Educ. Technol. 31, 542–553

[104] Pires K and Simon G (2015) YouTube live and Twitch: a tour of user-generated live streaming systems. In Proceedings of the 6th ACM Multimedia Systems Conference, Portland, pp. 225–230, ACM

[105] Cullinan J (2021) The disconnected: COVID-19 and disparities in access to quality broadband for higher education students. Int. J. Educ. Technol. High. Educ. 18, 26 34778524 10.1186/s41239-021-00262-1 PMC8137268

[106] Hsu C-H (2013) Biocloud: cloud computing for biological, genomics, and drug design. Biomed. Res. Int. 2013, 909470

[107] Langmead B and Nellore A (2018) Cloud computing for genomic data analysis and collaboration. Nat. Rev. Genet. 19, 208–219 29379135 10.1038/nrg.2017.113 PMC6452449

[108] Pouliakis A (2014) Cloud computing for biolabs. In Cloud Computing Applications for Quality Health Care Delivery (Moumtzoglou A and Kastania A, eds), pp. 228–249, IGI Global

[109] Yaacoub E and Alouini M-S (2020) A key 6G challenge and opportunity – connecting the base of the pyramid: a survey on rural connectivity. Proc. IEEE 108, 533–582

[110] Zhang C (2021) On telecommunication service imbalance and infrastructure resource deployment. IEEE Wirel. Commun. Lett. 10, 2125–2129

[111] Zhang C (2022) Big communications: connect the unconnected. Front. Comms. Net. 3, 785933

[112] Gerber LC (2016) Interactive biotechnology: design rules for integrating biological matter into digital games. In Proceedings of DiGRA/FDG 2016 Conference, pp. 1–16, DiGRA

[113] Lee SA and Riedel-Kruse IH (2022) Micro-HBI: human–biology interaction with living cells, viruses, and molecules. Front. Comp. Sci. 4, 849887

[114] Pataranutaporn P (2020) Living bits: opportunities and challenges for integrating living microorganisms in human-computer interaction. In Proceedings of the Augmented Humans International Conference, article 30, ACM

[115] Sharp P (2015) International IACUCs and outside collaborations. In The Care and Feeding of an IACUC. The Organization and Management of an Institutional Animal Care and Use Committee (2nd edn) (Petrie WK and Wallace SL, eds), pp. 185–202, CRC Press

[116] Naicker A (2022) Collaborative online international learning (COIL): preparedness and experiences of South African students. Innov. Educ. Teach. Int. 59, 499–510

[117] Ahmed T (2024) Large-scale and versatile deployment of biology cloud labs in schools through teacher driven curricula design. In Proceedings of the Eleventh ACM Conference on Learning@Scale, pp. 524–529, ACM

[118] Cybulski JS (2014) Foldscope: origami-based paper microscope. PLoS One 9, e98781 24940755 10.1371/journal.pone.0098781 PMC4062392

[119] Clark J (2000) Extended stability of restriction enzymes at ambient temperatures. BioTechniques 29, 536–542 10997268 10.2144/00293st06

[120] Sypsas A and Kalles D (2018) Virtual laboratories in biology, biotechnology and chemistry education: a literature review. In PCI’18: Proceedings of the 22nd Pan-Hellenic Conference on Informatics, pp. 70–75, ACM

[121] Rimmer M (2023) The UNDP accelerator lab network. In Intellectual Property Rights in the Post Pandemic World (Pihlajarinne T , eds), pp. 246–276, Edward Elgar Publishing

[122] Carlone HB and Johnson A (2007) Understanding the science experiences of successful women of color: Science identity as an analytic lens. J. Res. Sci. Teach. 44, 1187–1218

[123] Chen S and Wei B (2022) Development and validation of an instrument to measure high school students’ science identity in science learning. Res. Sci. Educ. 52, 111–126

[124] Bliss SS (2023) Learning and STEM identity gains from an online module on sequencing-based surveillance of antimicrobial resistance in the environment: an analysis of the PARE-Seq curriculum. PLoS ONE 18, e0282412 36897842 10.1371/journal.pone.0282412 PMC10004520

[125] Wang R (2022) Ultra-dense LEO satellite-based communication systems: a novel modeling technique. IEEE Comms. Mag. 60, 25–31

[126] Ye J (2021) Earth rotation-aware non-stationary satellite communication systems: modeling and analysis. IEEE Trans Wirel. Commun. 20, 5942–5956

[127] Zedini E (2020) Performance of multibeam very high throughput satellite systems based on FSO feeder links with HPA nonlinearity. IEEE Trans. Wirel. Commun. 19, 5908–5923

[128] Alsharoa A and Alouini M-S (2020) Improvement of the global connectivity using integrated satellite-airborne-terrestrial networks with resource optimization. IEEE Trans. Wirel. Commun. 19, 5088–5100

[129] Belmekki BEY (2024) Cellular network from the sky: toward people-centered smart communities. IEEE Open J. Comms. Soc. 5, 1916–1936

[130] Lou Z (2023) HAPS in the non-terrestrial network nexus: prospective architectures and performance insights. IEEE Wirel Comms. 30, 52–58

[131] Huang Q (2023) System-level metrics for non-terrestrial networks under stochastic geometry framework. ArXiv, Published online February 7, 2023. 10.48550/arXiv.2302.03376

[132] Javed S (2023) An interdisciplinary approach to optimal communication and flight operation of high-altitude long-endurance platforms. IEEE Trans. Aerosp. Electron. Syst. 59, 8327–8341

[133] Belmekki BEY and Alouini M-S (2022) Unleashing the potential of networked tethered flying platforms: prospects, challenges, and applications. IEEE Open J. Veh. Technol. 3, 278–320

[134] Zaid AA (2024) Aerial-aided mmWave VANETs using NOMA: performance analysis, comparison, and insights. IEEE Trans. Veh. Technol. 73, 4742–4758

[135] Kishk M (2020) Aerial base station deployment in 6G cellular networks using tethered drones: the mobility and endurance tradeoff. IEEE Veh. Technol. Mag. 15, 103–111

[136] El Falou A and Alouini M-S (2022) Enhancement of rural connectivity by recycling TV towers with massive MIMO techniques. IEEE Comms. Mag. 61, 78–83

[137] Aji LS (2017) The adoption of TV white space technology as a rural telecommunication solution in Indonesia. In 2017 15th International Conference on Quality in Research (QiR): International Symposium on Electrical and Computer Engineering, pp. 479–484, IEEE

[138] Grissa M (2019) TrustSAS: a trustworthy spectrum access system for the 3.5 GHz CBRS band. In IEEE INFOCOM 2019: IEEE Conference on Computer Communications, pp. 1495–1503, IEEE

[139] Mehrpouyan H (2014) Improving bandwidth efficiency in E-band communication systems. IEEE Comms. Mag. 52, 121–128

[140] Trichili A (2020) Roadmap to free space optics. J. Opt. Soc. Am. B 37, A184–A201

[141] Jung K-J (2020) Unified finite series approximation of FSO performance over strong turbulence combined with various pointing error conditions. IEEE T Comms 68, 6413–6425

[142] Jeon H-B (2023) Free-space optical communications for 6G wireless networks: challenges, opportunities, and prototype validation. IEEE Commun. Mag. 61, 116–121

[143] Hrabowski FA (2011) Boosting minorities in science. Science 331, 125 21233350 10.1126/science.1202388

[144] Beuermann DW (2015) One laptop per child at home: short-term impacts from a randomized experiment in Peru. Am. Econ. J. Appl. Econ. 7, 53–80

[145] Cristia J (2017) Technology and child development: evidence from the one laptop per child program. Am. Econ. J. Appl. Econ. 9, 295–320

[146] Pollack Ichou R (2018) Can MOOCs reduce global inequality in education? Australas. Mark. J. 26, 116–120

